# Comparison of Two Types of Electrodes for Measuring Submental Muscle Activity During Swallowing

**DOI:** 10.7759/cureus.59726

**Published:** 2024-05-06

**Authors:** Yoshiaki Ihara, Hirotaka Kato, Atsumi Sunakawa, Kouzou Murakami, Akira Minoura, Kojiro Hirano, Yoshio Watanabe, Masaki Yoshida, Akatsuki Kokaze, Yoshinori Ito

**Affiliations:** 1 Department of Oral Health Management, Division of Oral Functional Rehabilitation Medicine, Showa University School of Dentistry, Tokyo, JPN; 2 Department of Oral Rehabilitation Medicine, Showa University Graduate School of Dentistry, Tokyo, JPN; 3 Department of Radiology, Division of Radiation Oncology, Showa University School of Medicine, Tokyo, JPN; 4 Department of Hygiene, Public Health and Preventive Medicine, Showa University School of Medicine, Tokyo, JPN; 5 Department of Otorhinolaryngology Head and Neck Surgery, Showa University School of Medicine, Tokyo, JPN; 6 Department of Medicine, Division of Respiratory Medicine and Allergology, Showa University School of Medicine, Tokyo, JPN; 7 Faculty of Health Sciences, Osaka Electro-Communication University, Osaka, JPN

**Keywords:** waveform analysis, swallowing, submandibular, surface electromyographic, rising time

## Abstract

Purpose: This study aimed to investigate the potential of a newly developed small electrode to accurately record muscle activity during swallowing.

Material and methods: This study included 31 healthy participants. The participants underwent swallowing trials with three types of material. The recordings involved the following conditions: 1) swallowing saliva, 2) swallowing 3 mL water, and 3) swallowing 5 mL water. Two types of electrodes, a conventional electrode (CE) and a newly developed small electrode (NE), were symmetrically positioned on the skin over the suprahyoid muscle group, starting from the center. From the surface electromyography data, the swallowing duration (s), peak amplitude, and rising time (duration from swallowing onset to peak amplitude: s) were measured. Additionally, the equivalence of characteristics of the waveform of muscle activities was calculated by using the variance in both the upper and lower confidence limits in duration and rising time.

Results: No significant differences in baseline, swallowing duration or rising time between the CE and NE were observed for any swallowing material. The peak amplitude was significantly higher for the NE than for the CE for all swallowing materials. The CE and NE displayed no significant difference in the equivalence of characteristics of the waveform of muscle activities for any swallowing material.

Conclusions: The gold-plated small electrodes utilized in this study indicated the ability to record the same characteristics of muscle activity as conventional electrodes. Moreover, it was able to capture the muscle activity of each muscle group with improved sensitivity in a narrow area, such as under the submandibular region, with more precision than that of conventional electrodes.

## Introduction

Swallowing is a complex sensorimotor phenomenon that involves several muscles. It comprises voluntary and involuntary contractions of up to 26 muscles [1,2]. Among the muscles related to swallowing, the suprahyoid muscles play an important role [3]. Dysphagia, a swallowing dysfunction, is associated with various diseases, such as head and neck cancer, stroke, and neuromuscular disorders [4]. The occurrence of dysphagia is high among patients with neuromuscular diseases, such as amyotrophic lateral sclerosis (35%) [5], stroke (11%-50%) [6-7], Parkinson’s disease (over 80%) [8], head and neck cancer (60%-75%) [9], trauma [10] and some modalities of treatment on head and neck region [11].

Videofluoroscopic swallowing study (VFSS) and fiberoptic endoscopic evaluation of swallowing (FEES) are widely used as gold standards for dysphagia management. Both examinations are effective in diagnosing dysphagia. However, VFSS exposes patients to radiation through fluoroscopic procedures, and FEES is invasive, causing patients to experience discomfort during nasal endoscopic procedures [12,13]. In contrast, surface electromyography (sEMG) is relatively noninvasive and provides valuable information about the muscles involved in the swallowing process, therefore, evaluating swallowing function [14,15].

A previous study reported by Hara et al. indicated that patients with dysphagia exhibited lower muscle activity in the suprahyoid muscle group, compared with healthy individuals in Japan [16]. That sEMG study on swallowing used two types of electrodes. The first type was a disk electrode in a bipolar configuration, which has been reported to effectively remove common noise and exhibit spatial filtering effects in addition to the tissue volume conductor’s low-pass filtering effect [17]. The second type was a concentric ring electrode (CRE), which is proposed to enhance surface bipolar recordings of bioelectric signals [17]. Recent studies on sEMG signals obtained during swallowing from the submental area, aiming for portable and remote monitoring of the swallowing movement, have focused on materials and fabrication procedures [18,19].

In swallowing rehabilitation, numerous studies have employed sEMG [20-22], including reports on tongue movement and neck muscle fatigue [23]. However, most previous studies used EMG recordings from individual muscles or muscle pairs to investigate muscle activity during swallowing. Considering that swallowing involves multiple muscles and cranial nerves, sEMG signals from only a few sparse sensors may not physiologically provide sufficient dynamic properties of the entire swallowing procedure. The electrodes used in these studies were too large for a detailed evaluation of muscle activity in the submandibular area, and they did not allow for the assessment of overlapping muscles such as the digastric, geniohyoid, and mylohyoid muscles.

Therefore, we hypothesized that the use of smaller electrodes would enable more detailed analyses of complex swallowing movements by analyzing detailed muscle activity in each submandibular region. This study aimed to investigate whether a newly developed 2-mm electrode could effectively record muscle activities similar to those recorded by existing electrodes during submandibular swallowing movement.

## Materials and methods

Participants

In this study, we did not perform a power analysis. Previous reports have shown no sex-related differences when measuring muscle activity in healthy adults [24]. Thus, in this study, 31 healthy volunteers (16 men and 15 women) with a mean age of 28.77 ± 2.49 years (average age ± standard deviation; SD) participated. Before data collection, participants were confirmed to have no temporomandibular, occlusal, swallowing, or mastication abnormalities. All participants were aged > 18 years. No other inclusion criteria were applied in this study. All participants provided written informed consent. This study was approved by Showa University Research Ethics Review Board (approval 21-088-A) and was conducted according to the Declaration of Helsinki.

Recordings

Two types of electrodes were used to assess muscle activity. The first electrode was a conventional electrode (CE) (G210C; Nihon Kohden, Tokyo, Japan). The second device was a newly developed small electrode (NE) (Okuchy, Tokyo, Japan). This NE featured a spherical contact surface with the body and was gold-plated. The NE used in this study was made of a sulfur composite steel material with gold plating. Both electrodes had a 2-mm diameter and were fixed to a 0.1-mm thick silicone rubbery sheet using a male thread as a mooring structure, secured with a corresponding nut.

**Figure 1:**
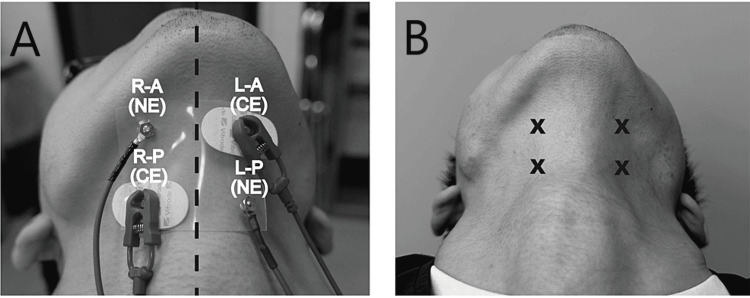
Position of electrode attachment A: Positions of the conventional type of electrode: L-A and R-P. Positions of newly developed small electrodes: L-P and R-A. B: X indicates the placement of the tips of electrodes. L: left, R: right, A: anterior, P: posterior, CE: conventional type of electrode, NE: newly developed small electrode

The two types of electrodes were symmetrically positioned on the skin over the suprahyoid muscle group, starting from the center (Figure 1A).

Additionally, the tips of electrodes were also placed in symmetrical positions over the suprahyoid muscle group (Figure 1B). All measurements were conducted by the same investigator (dentist). For the analysis, the average of the values obtained from the corresponding electrodes on both sides was used. The recordings were conducted with the same participants in the same resting sitting position. Moreover, the recording was conducted to eliminate sEMG artifacts as much as possible.

Data recordings

The participants underwent swallowing trials with three types of materials, and the swallowing movements were recorded about each material three times. With reference to previous studies using sEMG to study swallowing, the recordings involved the following conditions in this study: 1) swallowing saliva, 2) swallowing 3 mL water, and 3) swallowing 5 mL water. The obtained sEMG muscle activity waveforms were integrated to analyze swallowing motion. For the sEMG analysis, the data were sampled at 500 Hz, and the raw signals underwent bandpass filtering (50-250 Hz), rectification, and integration (time constant = 50 ms). The data acquisition system used for the sEMG analysis was BIMUTAS-Video, developed by KISSEI COMTEC, Nagano, Japan.

**Figure 2:**
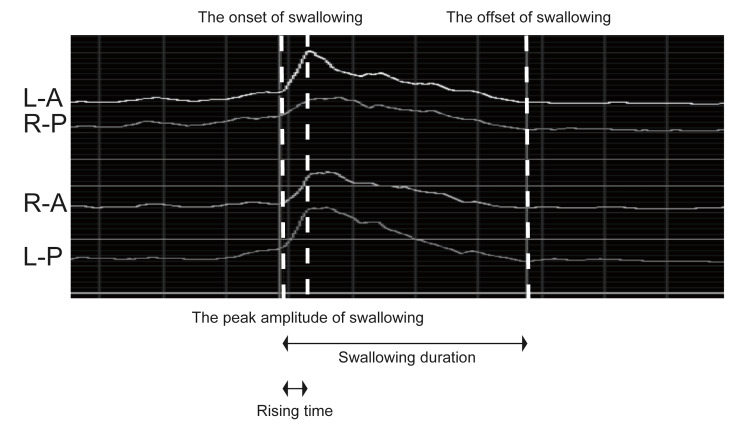
Measurements of surface electromyography (sEMG) data L: left, R: right, A: anterior, P: posterior. L-A, R-P: sEMG data from the conventional electrode. R-A, L-P: sEMG data from the newly developed small electrode. Onset of swallowing: point at which the sEMG signals increased abruptly from baseline. Offset of swallowing: point at which the sEMG signals returned to baseline. Peak amplitude of swallowing: highest amplitude reached during the action (mV). Swallowing duration: time between the onset and offset points for each measure (s). Rising time: duration between the onset of swallowing and time at peak amplitude.

From the sEMG data, several parameters were measured, including baseline (μV), swallowing duration (“duration,” measured in seconds: s), peak amplitude (“peak,” measured in μV), and rising time (the duration from swallowing onset to peak amplitude, measured in seconds: s). Additionally, the sEMG traces were evaluated to identify the onset and cessation of activity during swallowing (Figure 2).

Consistent with previous studies, the onset of swallowing was identified as the point where the sEMG signals exhibited a sudden increase from the baseline. The offset of swallowing was determined as the point where the sEMG signals returned to baseline [25]. The duration of swallowing represented the time between the onset and cessation points for each measure, whereas the peak represented the highest amplitude (activity) reached during the action potential’s duration. The rising time was the duration from the onset of swallowing to the time at peak amplitude, encompassing the interval between the onset and peak amplitude. In addition, the equivalence of waveform characteristics related to muscle activities was determined by assessing the variance in both upper and lower confidence limits within the duration and rising time of swallowing.

Procedures

Participants were instructed to sit comfortably in an upright position. The two types of electrodes (CE and NE) were placed on the skin over the suprahyoid muscle group. Subsequently, the participants were instructed to perform three types of swallowing: 1) swallowing saliva, 2) swallowing 3 mL of water, and 3) swallowing 5 mL of water. The water was administered orally using a syringe. Participants were instructed to hold the water in a supralingual position and then swallow it in a single motion to minimize sEMG variations caused by tongue movements during swallowing.

Data analyses

The sEMG data were statistically analyzed by averaging the values obtained from the left and right electrodes of the CE and NE in the three recordings. The sEMG results indicated a normal distribution and were subjected to a t-test. Additionally, the equivalence of the sEMG waveform between the CE and NE was determined based on the variance of the difference, using a 95% confidence interval for the duration and rising time of each swallowed material. In this study, we considered a p-value of < 0.05 as statistically significant.

## Results

Baseline sEMG activity

There were no significant differences in baseline sEMG activities between the CE and NE (CE; 0.09, SD = 0.08 mv, NE; 0.08, SD = 0.08 mv, p = 0.341).

Saliva Swallowing

The mean duration of saliva swallowing was 0.685 (SD = 0.166) s for the CE and 0.680 (SD = 0.176) s for the NE. No significant difference was observed between the CE and NE groups (p = 0.758). Similarly, the mean rising time of saliva swallowing for the CE was 0.256 (SD = 0.144) s and 0.246 (SD = 0.143) s for the NE. No significant difference was observed between the CE and NE groups (p = 0.395). However, the mean peak amplitude of saliva swallowing was 0.363 (SD = 0.229) mV for the CE and 0.436 (SD = 0.318) mV for the NE. The mean peak amplitude of the NE was significantly higher than that of the CE (p = 0.0002) (Table 1).

**Table 1:** Results of saliva swallowing Results of saliva swallowing, where CE stands for the conventional type of electrode, and NE represents the newly developed type of electrode. (**p < 0.01)

	CE	NE	p-value
Duration (s)	0.685 (SD = 0.166)	0.680 (SD = 0.176)	0.758
Rising time (s)	0.256 (SD = 0.144)	0.246 (SD = 0.143)	0.395
Peak amplitude (mv)	0.363 (SD = 0.229)	0.436 (SD = 0.318)	0.0002^**^

3-mL Water Swallowing

The mean duration of 3-mL water swallowing was 0.883 (SD = 0.204) s for the CE and 0.875 (SD = 0.209) s for the NE. No significant difference was observed between the CE and NE groups (p = 0.692). Similarly, the mean rising time of 3-mL water swallowing was 0.382 (SD = 0.196) s for the CE and 0.361 (SD = 0.198) s for the NE. Again, there was no significant difference between the CE and NE groups (p = 0.112). However, the mean peak amplitude of 3-mL water swallowing was 0.329 (SD = 0.229) mV for the CE and 0.436 (SD = 0.318) mV for the NE. The mean peak amplitude of the NE was significantly higher than that of the CE (p < 0.0001) (Table 2).

**Table 2:** Results of 3-mL water swallowing Results of 3-mL water swallowing, where CE stands for the conventional type of electrode, and NE represents the newly developed electrode type. (**p < 0.01)

	CE	NE	p-value
Duration (s)	0.883 (SD = 0.204)	0.875 (SD = 0.209)	0.692
Rising time (s)	0.382 (SD = 0.196)	0.361 (SD = 0.198)	0.112
Peak amplitude (mv)	0.329 (SD = 0.229)	0.436 (SD = 0.318)	< 0.0001^**^

5-mL Water Swallowing

The mean duration of 5-mL water swallowing was 0.902 (SD = 0.175) s for the CE and 0.887 (SD = 0.171) s for the NE. There was no significant difference between the CE and NE groups (p = 0.118). Similarly, the mean rising time of 5-mL water swallowing was 0.338 (SD = 0.163) s for the CE and 0.336 (SD = 0.178) s for the NE. Again, there was no significant difference between the CE and NE groups (p = 0.928). However, the mean peak amplitude of 5-mL water swallowing was 0.352 (SD = 0.152) mV for the CE and 0.407 (SD = 0.181) mV for the NE. The mean peak amplitude of the NE was significantly higher than that of the CE (p < 0.0001) (Table 3)

**Table 3:** Results of 5-mL water swallowing Results of 5-mL water swallowing, where CE stands for the conventional type of electrode, and NE represents the newly developed type of electrode. (**p < 0.01)

	CE	NE	p-value
Duration (s)	0.902 (SD = 0.175)	0.887 (SD = 0.171)	0.118
Rising time (s)	0.338 (SD = 0.163)	0.336 (SD = 0.178)	0.928
Peak amplitude (mv)	0.352 (SD = 0.152)	0.407 (SD = 0.181)	< 0.0001^**^

Equivalence of the sEMG waveform

Saliva Swallowing

No significant difference in the duration of saliva swallowing was indicated by the variance in both the upper and lower confidence limits (upper: p = 0.379; lower: p = 0.620). Similarly, for the rising time of saliva swallowing, no significant difference was indicated by the variance of difference in either the upper or lower confidence limit (upper: p = 0.198, lower: p = 0.802).

3-mL Water Swallowing

No significant difference in the duration of 3-mL water swallowing was indicated by the variance in both the upper (p = 0.346) and lower (p = 0.654) confidence limits. Similarly, for the rising time of 3-mL water swallowing, the variance of the difference indicated no significant difference in either the upper (p = 0.060) or lower (p = 0.944) confidence limit.

5-mL Water Swallowing

The variance of the difference in the duration of 5-mL water swallowing indicated no significant difference in either the upper (p = 0.069) and lower (p = 0.941) confidence limits. Similarly, for the rising time, the variance of the difference in 5-mL water swallowing also showed no significant difference in both the upper (p = 0.464) and lower (p = 0.536) confidence limits.

## Discussion

This study aimed to investigate whether a newly developed 2-mm electrode could effectively record muscle activities similar to those recorded by existing electrodes during submandibular swallowing movement. The results of this study indicate that the CE and NE were able to record the same muscle activities. There was no significant difference between the CE and NE in terms of the duration and rising time for any swallowed materials. However, the peak amplitude of each NE material was significantly higher than that of the CE. Therefore, the study’s findings suggest that the CE and NE could record the same waveform characteristics of swallowing, regardless of the material being swallowed.

Swallowing muscle activities, as measured by sEMG, can be influenced by surrounding muscles, with the impact being more significant in muscles closer to the surface [25]. In this study, the CE and NE electrodes were positioned in contrasting locations with different anteroposterior and posterior positions on the left and right sides. This variability might lead to differences in muscle activity recorded on the left and right sides. However, a previous report indicated that muscle activity values demonstrated good swallowing synchronization on both sides, as expected in healthy individuals [25]. Therefore, the results of the study, using the average of the values obtained from the left and right electrodes, suggest that there is minimal difference in muscle activity between the CE and NE on the left and right sides.

A previous study suggested that the swallowing duration varies depending on the bolus volume and consistency of the swallowed material [26]. This study observed a similar tendency, with the swallowing duration differing among the materials swallowed. Specifically, the swallowing duration of saliva was shorter than that of 3- and 5-mL water for both the CE and NE. However, no significant difference in swallowing duration was observed between the CE and NE for any swallowed material. This suggests that the CE and NE could record the same duration of swallowing movement for all materials, regardless of the properties of the electrode material.

The rising time of swallowing has been examined in numerous studies using sEMG. Previous studies have shown that the duration of swallowing varies depending on the amount and viscosity of the material swallowed [27-29]. In this study, the rising time varied depending on the swallowed material. Specifically, the rising time of saliva swallowing was shorter than that of 3- and 5-mL water swallowing; however, there was no significant difference in the rising time between 3- and 5-mL water swallowing. As the participants in this study were healthy, young adults who did not require additional effort to swallow, potential influences on swallowing duration were minimized. Oral and pharyngeal swallowing duration typically increases in older individuals [30]. Consequently, no significant difference was observed in the amount of water used.

The peak amplitude of the NE was significantly higher than that of the CE under all conditions. This difference is likely due to variations in the electrode material.

The surface material yielded one of the most significant differences between the CE and NE. The surface material of the CE was silver chloride, whereas the NE’s surface material was gold. Gold and platinum are polarizable electrodes that exhibit strong polarization properties because charge transfer on their surfaces is less likely to occur. In contrast, silver chloride electrodes are considered non-polarizable electrodes because of their low electrical resistance, resulting in low electrode resistance and polarization. A previous study found that electromyographic activity was influenced by the electrode materials because of the difference in conductivity and skin preparation for the monitoring of the masseter muscle [31]. Consequently, although there were differences in the recording region, the NE, using gold, recorded larger signals for the same muscle activity.

Moreover, previous reports have demonstrated that the electromyogram obtained is influenced by electrode shape, leading to noise interference [32]. Another study reported that signal amplification increased with greater distance between the ends of the electrodes [33]. The CE electrode dimensions were 18 mm × 36 mm. In contrast, the NE was much smaller, at 2 mm. In this study, the electrodes were positioned in relation to the center, potentially resulting in increased distance between the electrodes in the NE, which is smaller in size than the CE. In previous study, it was reported that the inter-electrode distance had significant effects on sEMG activities [34]. Thus, the difference of the inter-electrode distance between NE and CE might be one of the reasons for the larger maximum amplitude recorded using the NE compared with that recorded using the CE.

A previous study compared the waveforms obtained from different muscles during the same task [35]. However, no study has reported waveforms recorded during the same task using electrodes made of different materials or examined the similarity of the waveforms. In this study, there were no significant differences in the duration and rising time between the CE and NE; however, a significant difference was observed in the mean peak amplitude of the waveform. In a previous report, the duration, rising time, and peak amplitude were compared for posture-induced changes in swallowing function in healthy individuals, and the swallowing function was analyzed using electromyography [36]. The results of this study show that although there were differences in the peak amplitude depending on the electrode material, there were no significant differences in other parameters in any of the recordings. This suggests that it is possible to record waveform changes during the same muscle activity. The results of this study indicate that there was no significant difference in the baseline between the CE and NE. This may suggest that the use of an NE will allow for a more sensitive recording of muscle activity. Additionally, the CE and NE were capable of capturing comparable waveforms of muscle activities during swallowing. Nevertheless, the NE displayed the potential for higher sensitivity, compared with the CE. Additionally, with its mere 2-mm diameter, the NE holds promise for precise recording of localized movements, particularly in the submandibular region where multiple muscles are present. This underscores the potential of utilizing NE-based sEMG to investigate the detailed movements of individual swallowing-related muscles in a non-invasive manner, distinguishing it from the VFSS and FEES approaches.

Although VFSS provides a comprehensive view of swallowing motion and FEES is particularly adept at observing the pharyngeal phase, NE-based sEMG holds the promise of comprehensively examining muscle activities from mastication to swallowing, encompassing insights into muscle coordination. With further research advancements, the prospective applications in clinical contexts may extend beyond biofeedback.

A CE is disposable and inexpensive in terms of cost, whereas an NE, although expensive, can be used repeatedly. In biofeedback using electromyographs, electrodes are used frequently; the advantage of repeated use of NEs is considered to be significant. In addition, the use of gold, a metal with stable surface properties, ensures stable measurements, even after repeated use.

Limitations

In this study, the identity of the waveforms was examined using the duration and rising time but only in a preliminary manner. Therefore, further investigations using waveform analysis methods, such as fast Fourier transform, are necessary to explore this aspect thoroughly in the future. Additionally, the electromyogram obtained was not compared with the actual tongue movements or other movements. Thus, recording actual movements using the VFSS and examining the relationship between recorded muscle activities and real movements is essential for future research. Additionally, we did not perform a sample size calculation or power analysis; the low sample size may be a potential limitation. Moreover, there were only a few young healthy participants. To enhance the generalizability of the present study's findings, future research should encompass a broader population by expanding the sample size and including participants across different age ranges. Impedance characteristics at each electrode were not examined in this study. Impedance characteristics have a significant impact on the results of this study and should be examined in the future. In this study, we did not investigate about occlusal vertical dimension of participants. All participants in this study were healthy adults, and they did not have any mastication abnormalities. However, it was reported that occlusal vertical dimension had significant effect on sEMG activities on swallowing [37]. Thus, it would be necessary to consider about occlusal vertical dimension of participants in future study.

## Conclusions

The gold-plated small electrodes utilized in this study indicated to be able to record same characteristic of muscle activity to conventional electrode. Moreover, it was able to capture the muscle activity of each muscle group with improved sensitivity in a narrow area, such as under the submandibular region, with more precision than that with conventional electrodes. Therefore, they are considered valuable for recording muscle activity in this specific region.

## References

[1] Hiramatsu T, Kataoka H, Osaki M, Hagino H (2015). Effect of aging on oral and swallowing function after meal consumption. Clin Interv Aging.

[2] Muhle P, Wirth R, Glahn J, Dziewas R (2015). [Age-related changes in swallowing. Physiology and pathophysiology]. Nervenarzt.

[3] Iida T, Tohara H, Wada S, Nakane A, Sanpei R, Ueda K (2013). Aging decreases the strength of suprahyoid muscles involved in swallowing movements. Tohoku J Exp Med.

[4] Wilkinson JM, Codipilly DC, Wilfahrt RP (2021). Dysphagia: evaluation and collaborative management. Am Fam Physician.

[5] Jaradeh S (2006). Muscle disorders affecting oral and pharyngeal swallowing. GI Motil Online.

[6] Duncan S, Gaughey JM, Fallis R, McAuley DF, Walshe M, Blackwood B (2019). Interventions for oropharyngeal dysphagia in acute and critical care: a protocol for a systematic review and meta-analysis. Syst Rev.

[7] Mann G, Hankey GJ, Cameron D (2000). Swallowing disorders following acute stroke: prevalence and diagnostic accuracy. Cerebrovasc Dis.

[8] Smithard DG, O'Neill PA, Parks C, Morris J (1996). Complications and outcome after acute stroke. Does dysphagia matter?. Stroke.

[9] Suttrup I, Warnecke T (2016). Dysphagia in Parkinson's disease. Dysphagia.

[10] Malagelada JR, Bazzoli F, Boeckxstaens G (2015). World gastroenterology organisation global guidelines: dysphagia--global guidelines and cascades update September 2014. J Clin Gastroenterol.

[11] Martin-Harris B, Jones B (2008). The videofluorographic swallowing study. Phys Med Rehabil Clin N Am.

[12] Wilson RD, Howe EC (2012). A cost-effectiveness analysis of screening methods for dysphagia after stroke. PM R.

[13] Vaiman M, Eviatar E (2009). Surface electromyography as a screening method for evaluation of dysphagia and odynophagia. Head Face Med.

[14] Roldan-Vasco S, Restrepo-Agudelo S, Valencia-Martinez Y, Orozco-Duque A (2018). Automatic detection of oral and pharyngeal phases in swallowing using classification algorithms and multichannel EMG. J Electromyogr Kinesiol.

[15] Hara K, Tohara H, Minakuchi S (2018). Treatment and evaluation of dysphagia rehabilitation especially on suprahyoid muscles as jaw-opening muscles. Jpn Dent Sci Rev.

[16] Farina D, Cescon C (2001). Concentric-ring electrode systems for noninvasive detection of single motor unit activity. IEEE Trans Biomed Eng.

[17] Lee Y, Nicholls B, Sup Lee D, Chen Y, Chun Y, Siang Ang C, Yeo WH (2017). Soft electronics enabled ergonomic human-computer interaction for swallowing training. Sci Rep.

[18] Kim MK, Kantarcigil C, Kim B (2019). Flexible submental sensor patch with remote monitoring controls for management of oropharyngeal swallowing disorders. Sci Adv.

[19] Huckabee ML, Cannito MP (1999). Outcomes of swallowing rehabilitation in chronic brainstem dysphagia: a retrospective evaluation. Dysphagia.

[20] Battel I, Walshe M (2023). An intensive neurorehabilitation programme with sEMG biofeedback to improve swallowing in idiopathic Parkinson's disease (IPD): a feasibility study. Int J Lang Commun Disord.

[21] Archer SK, Smith CH, Newham DJ (2021). Surface electromyographic biofeedback and the effortful swallow exercise for stroke-related dysphagia and in healthy ageing. Dysphagia.

[22] Furutera H, Kawakami S, Kodama N, Manda Y, Kitagawa K, Nakahara R, Minagi S (2021). Detection of muscle fatigue caused by repeated posterior tongue lift movement from neck surface EMG: a pilot study. J Oral Rehabil.

[23] van den Engel-Hoek L, de Groot IJ, Esser E, Gorissen B, Hendriks JC, de Swart BJ, Geurts AC (2012). Biomechanical events of swallowing are determined more by bolus consistency than by age or gender. Physiol Behav.

[24] Leow LP, Huckabee ML, Sharma S, Tooley TP (2007). The influence of taste on swallowing apnea, oral preparation time, and duration and amplitude of submental muscle contraction. Chem Senses.

[25] Castroflorio T, Deregibus A, Bargellini A, Debernardi C, Manfredini D (2014). Detection of sleep bruxism: comparison between an electromyographic and electrocardiographic portable holter and polysomnography. J Oral Rehabil.

[26] Nascimento WV, Cassiani RA, Santos CM, Dantas RO (2015). Effect of bolus volume and consistency on swallowing events duration in healthy subjects. J Neurogastroenterol Motil.

[27] Newman R, Vilardell N, Clavé P, Speyer R (2016). Effect of bolus viscosity on the safety and efficacy of swallowing and the kinematics of the swallow response in patients with oropharyngeal dysphagia: white paper by the European Society for Swallowing Disorders (ESSD). Dysphagia.

[28] Kumagai H, Hasegawa-Tanigome A, Ninomiya K, Yamaguchi Y, Kumagai H (2021). Physical and textural properties of foods with swallowing ease for aged people. Food Sci Technol Res.

[29] Perlman AL, Palmer PM, McCulloch TM, Vandaele DJ (1999). Electromyographic activity from human laryngeal, pharyngeal, and submental muscles during swallowing. J Appl Physiol (1985).

[30] Ding R, Logemann JA, Larson CR, Rademaker AW (2003). The effects of taste and consistency on swallow physiology in younger and older healthy individuals: a surface electromyographic study. J Speech Lang Hear Res.

[31] Prasad S, Farella M, Paulin M, Yao S, Zhu Y, van Vuuren LJ (2021). Effect of electrode characteristics on electromyographic activity of the masseter muscle. J Electromyogr Kinesiol.

[32] Barrera CS, Piña-Martínez E, Roberts R, Rodriguez-Leal E (2022). Impact of size and shape for textile surface electromyography electrodes: a study of the biceps brachii muscle. Text Res J.

[33] Merletti R, Muceli S (2019). Tutorial. Surface EMG detection in space and time: best practices. J Electromyogr Kinesiol.

[34] Afsharipour B, Soedirdjo S, Merletti R (2019). Two-dimensional surface EMG: the effects of electrode size, interelectrode distance and image truncation. Biomed Signal Process Control.

[35] Ye-Lin Y, Prats-Boluda G, Galiano-Botella M, Roldan-Vasco S, Orozco-Duque A, Garcia-Casado J (2022). Directed functional coordination analysis of swallowing muscles in healthy and dysphagic subjects by surface electromyography. Sensors (Basel).

[36] Shiino Y, Sakai S, Takeishi R (2016). Effect of body posture on involuntary swallow in healthy volunteers. Physiol Behav.

[37] MacAvoy SK, Jack HC, Kieser J, Farella M (2016). Effect of occlusal vertical dimension on swallowing patterns and perioral electromyographic activity. J Oral Rehabil.

